# Challenges in the Management of Type 1 Diabetes Mellitus (T1DM) in a Pediatric Patient Due to Social Determinants of Health: A Case Report

**DOI:** 10.7759/cureus.39306

**Published:** 2023-05-21

**Authors:** Crystal Gianvecchio, Jose Cabas, Delanie Perez, Katherine Semidey

**Affiliations:** 1 Pediatrics, Florida International University, Herbert Wertheim College of Medicine, Miami, USA

**Keywords:** case report, uninsured patients, low-income patients, social determinants of health (sdoh), type 1 diabetes mellitus (t1dm), type 1 diabetes mellitus (t1d), pediatric case report

## Abstract

Type 1 diabetes mellitus is a rare pediatric condition that is complex to treat and requires careful life-long management. This report outlines a case of a pediatric patient who recently immigrated to the United States without financial resources or health insurance. The social determinants of health play a prominent role in this case as they have created barriers for the patient in obtaining insulin and maintaining adequate glycemic control. Pediatricians should be aware of how social determinants of health influence glucose management and be prepared to help their patients overcome barriers to parental education and treatment.

## Introduction

Type 1 diabetes mellitus (T1DM) is a rather rare diagnosis in children (30 cases per every 10,000 US children) and is a complex diagnosis to manage [1]. A thorough understanding of diabetic concepts like carbohydrate counting, sliding scales, potential complications, and management of hyperglycemia and hypoglycemia is necessary for proper glucose control and long-term survival. In the pediatric population, these difficulties become even more pronounced as young children cannot manage their glucose alone, requiring close monitoring, supervision, and assistance at home and at school. In addition to the complexity, it is also extremely expensive to treat. Insulin costs for an average adolescent can be near $300 per month, even when utilizing free discount prescription cards like GoodRx [2]. In low-income children without access to insurance, each one of these challenges becomes magnified and, therefore, incredibly difficult to overcome. Social determinants of health (SDOH) come front and center in these cases. Each barrier to care must be addressed which requires time, effort, patience, and knowledge of available resources.

## Case presentation

A five-year-old, uninsured male with T1DM who recently arrived from Colombia several months ago presented to the clinic with his mother for a follow-up of his diabetes management. His mother came in with concerns about his diminishing supply of insulin, consistently high blood glucose readings, and interest in obtaining diabetes care for the patient at school with a nurse.

Per the mother, the patient was diagnosed with T1DM at the age of two by an endocrinologist in Colombia. The patient has a relevant family history of his father also having a diagnosis of T1DM. Prior to the patient’s arrival in the United States, it was noted by his mother that his hemoglobin A1C was 8%. The patient had arrived in the United States with a small insulin supply which lasted him about two months. He was subsequently seen at a local children’s hospital when he ran out of insulin and his glucose spiked. The patient was admitted for four days, his glucose was stabilized, and a new insulin regimen was established. He was provided with a two-month supply of insulin upon discharge and told to follow up with his pediatrician. Given his lack of insurance and familiarity with the medical system, his family struggled to find low-cost follow-up outpatient care. At this visit, the patient had about two weeks’ worth of insulin remaining. Since discharge, his mother describes that the patient’s blood glucose readings in the morning range from 60 to 80 mg/dl. Typically around 10 A.M., before the patient has lunch, his mother receives a text from his school stating that his blood glucose readings are between 300 and 400 mg/dl. The school nurse does not administer insulin to him. Therefore, his mother makes the trip to the school to administer 2 units of Humalog (fast-acting insulin lispro) if his reading is in the 300 range and 3 units if his reading is in the 400 range. This has been occurring daily. For this reason, the school provided the mom with the referral for in-school nursing services forms to be completed. The mother does not keep a log of his blood glucose readings and is unaware of how to count carbs or use a sliding-scale insulin regimen. He also receives five units of Lantus (long-acting insulin glargine) at bedtime.

The patient’s immigration status, which leaves the family ineligible for insurance, and his social history are important to consider. The patient, his mother, and his ten-year-old brother arrived from Colombia two months prior to establishing care at the clinic. The family plans to apply for asylum in the coming months. The family lives in the patient’s aunt’s home, and the only individual working in the household is this aunt, who works at a nail salon. The aunt owns a car that she uses to get to and from work, limiting the patient’s ability to attend regular health appointments. Per mom, they are struggling to afford basic things like food and school supplies.

The patient has thus far not experienced any symptoms related to uncontrolled T1DM, including seizures, nausea, vomiting, increased frequency of urination, increased thirst, or blurred vision, and, according to the mother, has not had any episodes consistent with diabetic ketoacidosis [3]. On physical exam, there were no abnormal findings aside from poor dentition and dental caries.

The plan to obtain a secure insulin supply for this patient consisted of completing applications for free medications through the philanthropic arms of pharmaceutical companies [4-6]. For his glucose management, the children’s hospital diabetes management hotline provided at discharge was contacted, and his insulin regimen was adjusted based on the estimated blood glucose measurements from his mother. Given his mother’s low understanding of carb counting, he was given a simple correction plan that was as follows: 1 unit should be given at breakfast, lunch, and dinner. At breakfast and dinner, a sliding scale would be utilized as well, an additional half unit with every 50 mg/dL above 250 mg/dL with a max of 3 units total. His bedtime Lantus was also lowered to 4 units to improve his low morning blood glucose readings. This new regimen was written out clearly in Spanish, in a color-coded Word document, and printed for his mother. Glucose logs were created in Spanish and printed as well so his glucose levels could be better tracked. This was all carefully reviewed with his mother, and her understanding of the new sliding scale was confirmed. His forms authorizing a school nurse to give his insulin were also completed with guidance to minimize physical activity when his blood glucose readings are above 300 mg/dL to avoid ketosis. To provide this patient with long-acting nighttime insulin, other insulin options were explored. Patient assistance paperwork for Novo Nordisk, the producer of Tresiba (long-acting insulin degludec) which is FDA-approved for pediatric patients over age one, was completed on behalf of the patient in order to provide him with an alternative medication to Lantus [7]. To provide the patient with Humalog, which is FDA-approved for pediatric patients above the age of three, an application for Lilly Cares, a non-profit organization that provides a variety of insulin products to those with financial need, was also completed [8].

The patient was scheduled for a follow-up through telehealth in two weeks to check his blood glucose logs. The patient’s mother was asked to log morning and evening glucose levels to evaluate whether the new insulin regimen was effective in maintaining glycemic control. In a telehealth visit, the mother presented glucose logs, which continued to show uncontrolled levels of morning glucose and inconsistent logging of morning and evening levels as only eight values were reported instead of the expected 14 for a seven-day period. Morning glucose values remained low despite decreasing Lantus from 5 units to 4 units at nighttime, and further follow-up appointments revealed that the patient’s mother had not decreased the dosage of Lantus to 4 units as instructed. As for evening glucose readings, the reported values showed improvement with glucose readings lower than previously reported values of 300-400 mg/dL. The patient was scheduled to return to the clinic in two weeks with new glucose recordings for an in-person appointment to discuss possible changes to the current plan and evaluate new logs.

The in-person appointment revealed that the patient’s mother is now consistently recording the patient's glucose levels. The patient is now receiving management from a nurse at school, who is following his insulin sliding scale as directed without any incidents. Glucose logs showed improvement in the management of daytime glucose with values ranging from 100 to 170 mg/dL before lunch, provided by the school nurse, as well as improved bedtime glucose levels with values ranging from 100 to 250 mg/dL, reported by his mother. Despite reporting controlled insulin values throughout the day, morning glucose readings remain below the goal of 80-130 mg/dL, with reported values within the range of 50-90 mg/dL. After further discussion about the Lantus dosage with his mother, she revealed to not have lowered the Lantus evening dose from 5 units to 4 units as instructed in the previous visit a month prior.

By this time, the patient was approved by Lilly Cares to receive Humalog at no cost and will be receiving a stable supply of fast-acting insulin for up to a year. Tresiba was temporarily provided by the clinic until the patient was approved by Novo Nordisk Patient Assistance Program to receive Tresiba at no cost for up to a year. As of 2023, the patient receives a stable supply of long-acting and fast-acting insulin through patient assistance programs. Informed consent was received from the parents of the patient for publication purposes of this case report.

## Discussion

This case illustrates a gap in diabetes management for young children with T1DM who face significant barriers to regular access to insulin and healthcare. This patient’s situation also highlights the need for culturally sensitive and effective patient education about T1DM and the severity of the condition when left uncontrolled.

While major pharmaceutical companies frequently have patient assistance programs that account for the population of patients who are uninsured or who have difficulty accessing insulin, pediatric patients face additional barriers in not meeting age requirements. Sanofi has an age requirement of at least six years old to provide Lantus at no cost, as Lantus is only FDA-approved over the age of six [9]. To provide this patient with long-acting nighttime insulin, other insulin options were explored. Patient assistance paperwork for Novo Nordisk, the producer of Tresiba (long-acting insulin degludec) which is FDA-approved for pediatric patients over age one, was completed on behalf of the patient in order to provide him with an alternative medication to Lantus [8]. To provide the patient with Humalog, which is FDA-approved for pediatric patients above the age of three, an application for Lilly Cares, a non-profit organization that provides a variety of insulin products to those with financial need, was also completed [9]. It is important for pediatricians to understand the eligibility requirements of pharmaceutical patient assistance programs that typically involve residency status, insurance status, income verification, and age (Table 1).

**Table 1:** Selected requirements of U.S. patient assistant programs providing insulin medications for pediatric patients All information was gathered from the website for each patient assistance program [4-6].

Patient Assistance Program	Insulin Medication(s) Provided	Age Requirement	Immigration Status Requirement	Financial Eligibility
Sanofi	Lantus (insulin glargine)	≥ 6 years old	Resident of the U.S. or U.S. territories	≤ 400% FPL
	Admelog (insulin lispro)			
	Apidra (insulin glulisine)			
	Toujeo (insulin glargine)			
	Insulin glargine			
	Soliqua (insulin glargine and lixisenatide)			
Lilly Cares Foundation	Humalog (insulin lispro)	≥ 3 years old	Resident of the U.S. or U.S. territories	None
	BASAGLAR (insulin glargine)	≥ 6 years old		
NovoCare	Fiasp (insulin aspart)	≥ 2 years old	U.S. citizen or legal resident	≤ 400% FPL
	Levemir (insulin detemir)			
	NovoLog (insulin aspart)			
	Tresiba (insulin degludec)			

The recommended management of pediatric T1DM requires close monitoring of blood glucose levels and carbohydrate intake as well as meal planning, exercise planning, urine ketone testing as needed, and long-term care from an endocrinologist [10,11]. The pediatric patient population in particular requires regular reassessment of insulin regimens due to shifts in insulin sensitivity as the child physiologically and sexually matures [12]. Close management is not readily available for pediatric patients that are uninsured, low-income, lack reliable transportation, and face significant language and knowledge barriers that make patient education extremely difficult.

**Figure 1:**
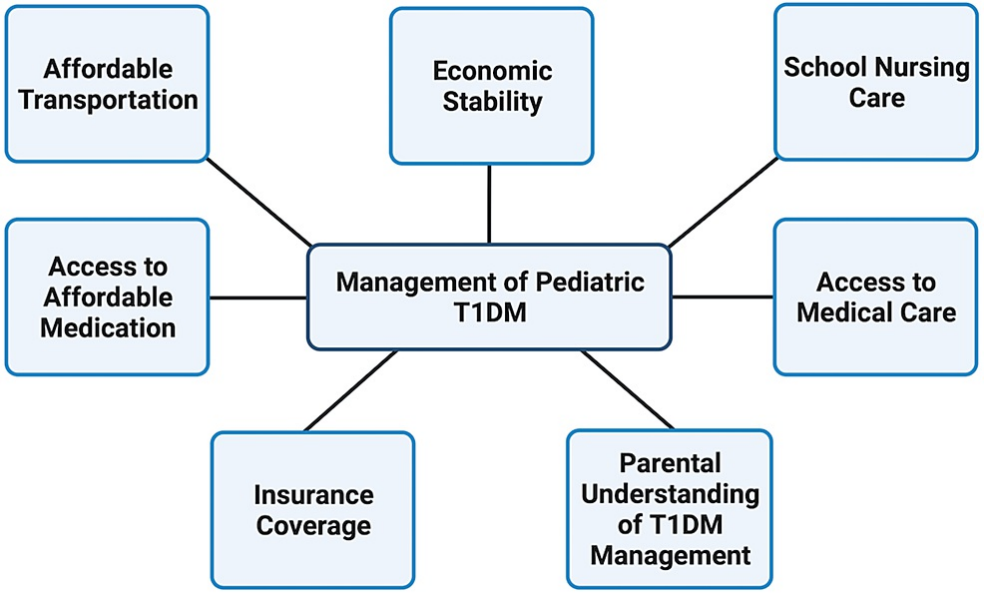
SDOH affecting management of T1DM in children

Patient education is paramount for the families of pediatric patients with T1DM as the patients are not able to monitor and regulate their blood glucose levels themselves. Importantly, patient education is also crucial in the event a pediatric patient has symptomatic hypoglycemia and requires the administration of glucagon (Figure 1). SDOH like poverty, fewer years of formal education, and identification with certain minority racial or ethnic groups such as Hispanic or Black have all been shown to correlate with lower degrees of parental patient literacy and less glycemic control in pediatric patients [13].

When pediatric patients have uncontrolled T1DM at a young age, they are at risk for short-term and long-term complications as well as increased mortality [14]. It has been shown that pediatric patients with T1DM are more likely to develop acute and chronic complications when they have concurrent socioeconomic factors that limit their ability to obtain the recommended treatment for their condition [15].

A temporary solution for this patient’s diminishing insulin supply, lack of patient education, and insulin regimen adjustment was found using a diabetes management line provided by the children’s hospital from which the patient was recently discharged; this is an invaluable resource that is not always available. Given this patient’s unstable immigration status, he is not eligible for free or low-cost insurance like Medicaid or Children’s Health Insurance Program and is not eligible for charity care from our local safety-net hospital. Hence, pediatricians should become well aware of not only patient assistance programs available for pediatric patients with T1DM but also local resources for pediatric endocrinology management especially those that can aid with patient education in multiple languages. T1DM is a lifelong condition that requires consistent management throughout childhood and adulthood and any lapse in care can cause significant alterations in the quality of life of patients, especially uninsured patients.

Taken as a whole, this case report presents how the SDOH can play a key role in disease management. It presents how factors such as immigration status, economic stability, parental understanding of the disease, insurance, and age can determine the chronic management of T1DM in pediatric patients. It also highlights possible solutions to address the factors preventing proper disease management. Implementing the use of sliding scale information in the spoken language of the patient’s family and helping patients apply to patient assistance programs can be applied by pediatricians throughout the United States. Some of the limitations presented in this case study are the lack of generalizability to other countries, as many assistance programs work within the United States. Another limitation is the variability of SDOH that is present for each patient. These factors may require creative solutions and it is up to physicians to address these barriers and find individualized solutions for each patient.

## Conclusions

T1DM is a rare pediatric condition that is complex to treat and requires careful life-long management. This case of a pediatric patient who recently immigrated to the United States without financial resources or health insurance highlights how the SDOH can make the management of T1DM even more difficult. Significant barriers arise in accessing insulin, understanding patient education about the condition, and exercising appropriate glycemic control. Knowledge of low-cost resources, patient assistance programs, creative solutions for parental education, and endocrinology services are critical for pediatric patients in similar situations who are at risk for serious complications and decreased quality of life if their condition is not properly managed at a young age.
